# Prevalence and molecular characterization of *Vibrio cholerae* from fruits and salad vegetables sold in Jakarta, Indonesia, using most probable number and PCR

**DOI:** 10.1186/s13104-022-05955-y

**Published:** 2022-03-03

**Authors:** Andrea Budiman, Kevin Kurnia, Diana E. Waturangi

**Affiliations:** Atma Jaya Catholic University of Indonesia, Faculty of Biotechnology, Jalan Raya Cisauk-Lapan No. 10, 15345 Tangerang, Banten Indonesia

**Keywords:** *Vibrio cholerae*, Fruits, Vegetables, MPN, Multiplex PCR

## Abstract

**Objective:**

Cholera is an intestinal infection caused by *Vibrio cholerae*, it is usually occurs in developing countries that lack of sanitation. In developing country including Indonesia, awareness importance of sanitation is still low. Unfortunately, research related to the detection of *V. cholerae* from fruit and vegetables in Indonesia is still rare. In this study, MPN method was used to determine the prevalence of *V. cholerae* followed by single and multiplex PCR to detect virulence genes, including *toxR, ctxA, tcpA*, *hlyA*, *ace*, *ompU*, and *zot*.

**Results:**

We found 3 fruits and 2 vegetables positive for *toxR* gene. Fruit samples which were showed *toxR* positive found from East Jakarta while for vegetables, it was recovered from West Jakarta and Central Jakarta. Twenty-three isolates were recovered from *toxR* positive samples. The result of antibiotic resistance analysis showed that 4.35% of the isolates resistant to gentamicin, streptomycin (17.39%), trimethoprim (52.17%), ciprofloxacin (30.43%), ampicillin (13.04%), nalidixic acid (82.61%), and polymyxin B (91.30%). None of these isolates were resistant to kanamycin. Combination of MPN and Multiplex PCR method can be used to detect the prevalence and characterize the virulence properties of *V. cholerae*.

## Introduction

Cholera is an infection in the small intestine caused by *Vibrio cholerae.* The main symptoms of cholera are vomiting and diarrhea that lead to dehydration and electrolyte imbalance. According to WHO (2015) [1], the incidence rate of cholera in Jakarta, Indonesia reached 0.45 cases per 1,000 populations with a case fatality rate of 1%. In 1961, *V. cholerae* O1 biotype El Tor was emerged in Celebes (Sulawesi), Indonesia and spread to the other islands of Indonesia and other countries causing the seventh pandemic of cholerae. While the first six of cholera pandemics in 1899–1923 were caused by the classical strains [2]. Cholera is the second leading cause of death for children under the age of 5 and at least 120,000 deaths occurred each year [3]. *V. cholerae* is a Gram-negative bacteria, facultative anaerobe, oxidase-positive, and have a single polar flagellum [4].

In small intestine, *V. cholerae* produces enterotoxins to disrupt ion transport and lead to dehydration [5]. The toxin production depends on the transcriptional activator ToxR that regulate cholera toxin gene expression (ctxA), TCP biogenesis (tcpA), outer membrane protein expression (ompU), and at least 17 distinct genes in O139 and O1 strains [6, 7]. There are many virulence factors that can cause cholera with different characteristics aside from cholera toxin, such as El Tor-like hemolysin (hlyA), a new CT, outer membrane protein (ompU), Shiga-like toxin (stx), and a zonula occludens toxin (zot) [8].

Conventional methods used to detect and characterize *V. cholerae* from samples are time-consuming, low sensitivity, and laborious. Furthermore, these methods are not efficient for the screening of a large number of samples [9]. Therefore, molecular methods are required for rapid and sensitive detection of *V. cholerae* by using specific primers associated with virulence genes. The objective of this research is to provide rapid and reliable method of detection of *V. cholerae* from salad vegetables and fruits. Also, to determine the prevalence and detection of virulence genes of *V. cholerae*.

## Main text

### Methods

#### Samples collection and handling

A total of 133 samples (57 fruits and 76 vegetables) were collected from several traditional markets and supermarkets in Jakarta from July 2012 until March 2013. We collected 10 fruits and 10 vegetables for each districts (North, South, West, East, and Central of Jakarta) to represent the data for Jakarta region. Fruits that could be eaten without peeling and vegetables that often eaten raw were selected for this study. Apple—*Malus domestica*, Star fruit—*Averrhoa carambola*, Rose apple—*Syzygium jambos*, Guava—*Psidium guajava*, Pear—*Pyrus L.*, Tomato—*Solanum lycopersicum*, Grape—*Vitis vinifera*, Bean sprout—*Vigna radiata*, Black nightshade—*Solanum nigrum*, Cabbage—*Brassica oleracea*, Carrot—*Daucus carota*, Chayote—*Sechium edule*, Coriander—*Coriandrum sativum*, Cucumber—*Cucumis sativus*, Lemon basil—*Ocimum citriodorum*, Lettuce—*Lactuca sativa*, Thai eggplant—*Solanum melongena*, Pea Shoot—*Pisum sativum*, Ulam raja—*Cosmos caudatus*, Watercress—*Nasturtium officinale*, Yard longbean—*Vigna unguiculata*. Samples were processed maximum 24 h after purchasing from the vendors.

#### Bacteria enumeration from samples

From each samples, about 5 g of cortex were cut and crushed. Then, it were inoculated to 25 ml of Alkaline Peptone Water (APW) medium pH 8.5 (Oxoid, England) and vortexed for 1 min. For enumeration, we used 3 tube of Most Probable Number (MPN) method. There were 9 tubes for each samples with dilution of 10^–1^, 10^–2^, and 10^–3^ which were divided equally. Nine MPN tubes of 10^–1^ dilution were added into 1 ml of solutions and vortexed for 1 min. Subsequently, 1 ml of samples were taken from 10^–1^ dilution and mixed with 10^–2^ dilution and so on until reached 10^–3^ dilution [6]. MPN tubes were incubated at *V. cholerae* optimum condition 37 °C and 120 rpm for 24 h.

#### DNA templates preparation

DNA template for single PCR was prepared from positive MPN tubes. One ml of the cultures were centrifuged 12,000×*g* for 5 min. Then, the supernatant was taken and resuspended in 200 µl of ddH2O, and boiled for 10 min [10]. The Supernatant was used as DNA template and stored at − 20 °C until further used.

#### Uniplex and Multiplex PCR

Uniplex PCR were used to detect the presence of *toxR* gene [11] from *V. cholerae* suspected samples. *V. cholerae* 3.21 (Atma Jaya culture collection) were used as positive control. The Total of PCR reaction was 25 μl containing of 12.5 μl of GoTaq green master mix (400 μM dNTP) (Promega, USA), 1 μl (10 μM) of each *toxR* forward and reverse primers, 2 μl of DNA template, and 8.5 μl of nuclease free water. PCR reaction was performed in a thermal cycler (Applied Biosystems, USA) with initial denaturation at 94 °C for 2 min, followed by 25 cycles consisting of 94 °C for 1 min, 62 °C for 1 min, and 72 °C for 1 min, and a post-extension step 72 °C for 10 min.

Multiplex PCR were used to detect other virulence-associated genes such as*, ctxA, tcpA, hlyA, ace, ompU*, and *zot* [8, 11]. The PCR mixture consisted of 25 μl of GoTaq green master mix, 1 µl of each primer pairs, 2.5 μl of DNA template, and nuclease free water until 50 µl of mixture. The final concentration used for the *toxR* primer was 50 µM; 16 µM for *ctxA* and *ompU* primer; 30 µM for *zot, ace, hlyA*, and *tcpA* primer. PCR condition were initial denaturation at 94 °C for 2 min followed by 20 cycles consisting of 94 °C for 1 min, 62 °C for 1 min, and 72 °C for 1 min, 10 cycles consisting of 94 °C for 1 min, 54 °C for 1 min, and 72 °C for 1 min, then post-extension step 72 °C for 10 min, and hold at 4 °C.

The PCR products were loaded on 1% (w/v) agarose gel with 1 × Tris Acetic EDTA (TAE) buffer and run with condition of 60 V for 150 min, and visualized under UV light after stained with ethidium bromide (EtBr).

#### Isolation of *V. cholerae* suspect

All of *V. cholerae* suspected colonies were selected from Thiosulphate Citrate Bile-salt Sucrose (TCBS) agar and grown at BHIA medium overnight. These colonies were tested with oxidase reagent 1% (w/v) solution of NNNN Tetra methyl-p-Phenylendiammonium dichloride (Merck). *V. cholerae* sh positive result (form purple colour after 30 s) for oxidase test on filter paper [6]. Afterwards, fresh positive culture from agar were taken with toothpick and stirred with 200 µl ddH2O until homogenized.

#### Serological and antibiotic resistance assay

Recovered *V. cholerae* from samples were refreshed into BHIA medium and incubated at 37 °C overnight and continued with serological assay using antiserum polyvalent O1 (Remel) and monovalent (Ogawa or Inaba) [6]. Firstly, the isolate was tested with polyvalent antiserum, if the result showed positive for serogroup O1, it will tested further with monovalent antiserum to determine whether it was serotype of Ogawa or Inaba. *V. cholerae* which were showed positive reaction with antiserum will form white clumps when it react with antiserum.

For antibiotic resistance assay, *V. cholerae* isolates were streaked on nutrient agar (NA) and incubated overnight at 37 °C. In this study, we used antibiotic discs such as polymyxin B (300 U), nalidixic acid (30 µg), trimethoprim (5 µg), ampicillin (10 µg), streptomycin (10 µg), gentamycin (10 µg), kanamycin (30 µg), and ciprofloxacin (5 µg) (Oxoid, Hampshire, England). Zone of inhibition was determined followed the guideline from Clinical and Laboratory Standard Institute (CLSI) [12].

### Results

A total of 99.25% of the samples (from 133 samples) showed positive results for MPN assay (Table 1). The prevalence rate of *V. cholerae* in fruits and vegetables obtained from 5 regions of Jakarta was 100%, except for fruits collected from South Jakarta (91.67%). A positive result means at least one MPN tube (out of 9 tubes) was turbid. The highest lower and upper number from MPN result was 420 MPN/ml and > 4100 MPN/ml respectively found in 36 samples fruits and 74 samples vegetables. The lowest lower and upper number was found from apple (Central Jakarta) and cabbage (North Jakarta) with value of 4.6 MPN/ml and 94 MPN/ml, respectively. Moreover, 3 fruits and 2 vegetables were positive for *toxR* gene detection.

**Table 1:** MPN results and presence of *toxR* gene for *V. cholerae* isolates from Jakarta, Indonesia region

Sample type	Source	Sample ID	*V. cholerae* level (MPN/ml) 95% confidence limit		*toxR* result by PCR	% *toxR* positive samples
			Lower	Upper		
Fruits	North Jakarta	TO.U.1	420	–	–	0
		TO.U.2	42	1000	–	
		AP.U.1	9	180	–	
		AP.U.2	42	1000	–	
		JB.U.1	42	1000	–	
		JA.U.1	420	–	–	
		PI.U.1	42	1000	–	
		TO.U.3	420	–	–	
		BL.U.1	420	–	–	
		TO.U.4	420	–	–	
	South Jakarta	BL.S.1	420	–	–	0
		TO.S.1	180	4100	–	
		PI.S.1	–	9.5	–	
		TO.S.2	18	420	–	
		BL.S.2	9	180	–	
		JA.S.1	420	–	–	
		JB.S.1	420	–	–	
		BL.S.3	420	–	–	
		TO.S.3	420	–	–	
		AP.S.1	18	420	–	
		PI.S.2	18	420	–	
		TO.S.4	420	–	–	
	West Jakarta	AP.B.1	18	420	–	0
		BL.B.1	420	–	–	
		JA.B.1	420	–	–	
		JB.B.1	420	–	–	
		TO.B.1	420	–	–	
		TO.B.2	420	–	–	
		JB.B.2	420	–	–	
		TO.B.3	420	–	–	
		BL.B.2	420	–	–	
		TO.B.4	420	–	–	
		PI.B.1	180	4100	–	
	East Jakarta	JA.T.1	420	–	–	21.4
		TO.T.1	420	–	–	
		BL.T.1	420	–	–	
		PI.T.1	420	–	–	
		AP.T.1	42	1000	–	
		BL.T.2	180	4100	–	
		JB.T.1	180	4100	–	
		PI.T.2	42	1000	–	
		AG.T.1	180	4100	–	
		AP.T.2	420	–	+	
		BL.T.3	420	–	–	
		PI.T.3	420	–	+	
		JB.T.2	420	–	–	
		BL.T.4	420	–	+	
	Central Jakarta	TO.P.1	420	–	–	0
		TO.P.2	420	–	–	
		AP.P.1	420	–	–	
		AP.P.2	4.6	94	–	
		PI.P.1	180	4100	–	
		TO.P.3	420	–	–	
		TO.P.4	420	–	–	
		AP.P.3	42	1000	–	
		BL.P.1	420	–	–	
		TO.P.5	420	–	–	
Vegetables	North Jakarta	SEL.U.1	420	–	–	0
		KOL.U.1	420	–	–	
		KET.U.1	420	–	–	
		KER.U.1	420	–	–	
		KEM.U.1	420	–	–	
		SEA.U.1	420	–	–	
		KET.U.2	420	–	–	
		WOR.U.1	420	–	–	
		KOL.U.2	4.6	94	–	
		KOL.U.3	420	–	–	
		KET.U.3	420	–	–	
		WOR.U.2	420	–	–	
	South Jakarta	SEL.S.1	420	–	–	0
		SEA.S.1	420	–	–	
		KET.S.1	420	–	–	
		KOL.S.1	420	–	–	
		SEL.S.2	420	–	–	
		KET.S.2	420	–	–	
		KOL.S.2	420	–	–	
		SEL.S.3	420	–	–	
		KET.S.3	420	–	–	
		KOL.S.3	420	–	–	
		SEL.S.4	420	–	–	
		KET.S.4	420	–	–	
		WOR.S.1	420	–	–	
		ULA.S.1	420	–	–	
		SEA.S.1	420	–	–	
		SEL.S.5	420	–	–	
		KEM.S.1	420	–	–	
		KOL.S.4	420	–	–	
	West Jakarta	KOL.B.1	420	–	–	5.26
		KOL.B.2	420	–	–	
		KET.B.1	420	–	–	
		SEL.B.1	420	–	–	
		KOL.B.3	420	–	–	
		KOL.B.4	420	–	–	
		KET.B.2	420	–	–	
		SEL.B.2	420	–	–	
		WOR.B.1	420	–	–	
		DOU.B.1	420	–	–	
		SEL.B.3	420	–	–	
		LAB.B.1	420	–	–	
		KET.B.3	420	–	–	
		KOL.B.5	420	–	–	
		WOR.B.2	420	–	+	
		KET.B.4	420	–	–	
		TAU.B.1	420	–	–	
		WOR.B.3	420	–	–	
		KOL.B.6	420	-	–	
	East Jakarta	SEL.T.1	420	––	–	0
		KEM.T.1	420	–	–	
		KOL.T.1	420	–	–	
		KEM.T.2	420	–	–	
		SEL.T.2	420	–	–	
		TER.T.1	420	–	–	
		KET.T.1	420	–	–	
		TER.T.2	420	–	–	
		LEU.T.1	420	–	–	
		TER.T.3	420	–	–	
		KET.T.2	420	-	–	
	Central Jakarta	KAC.P.1	420	–	–	6.25
		KEM.P.1	420	–	–	
		TER.P.1	420	–	–	
		KEM.P.2	420	–	–	
		KET.P.1	420	–	–	
		TAU.P.1	420	–	-	
		KOL.P.1	420	–	–	
		WOR.P.1	420	–	–	
		SEL.P.1	8.7	94	+	
		KOL.P.2	420	–	–	
		KOL.P.3	420	–	–	
		WOR.P.2	420	–	–	
		SEL.P.2	420	–	–	
		KET.P.2	420	–	–	
		KET.P.3	420	–	–	
		KOL.P.4	420	–	–	

*AP* Apple, *BL* Star fruit, *JA* Rose apple, *JB* Guava, *PI* Pear, *TO* Tomato, *AG* Grape, *TAU* Bean sprout, *LEU* Black nightshade, *KOL* Cabbage, *WOR* Carrot, *LAB* Chayote, *KER* Coriander, *KET* Cucumber, *KEM* Lemon basil, *SEL* Lettuce, *TER* Thai eggplant, *DOU* Pea Shoot, *ULA* Ulam raja, *SEA* Watercress, *KAC* Yard longbean

Total single colonies of *V. cholerae* recovered from positive samples were 23 colonies. These isolates recovered from apple (5), star-fruit (1), pear (7), carrot (2), and lettuce (8). Most of the isolates were found from East Jakarta (13 of 23; 56.52%). None of fruits sample from Central and West Jakarta was positive, as well as for vegetables, no positive result found from East Jakarta.

Most of the single isolates belong to the serogroup O1 (18 of 23; 78.26%) with 10 isolates belong to Ogawa serotype and 8 serotype of Inaba (Table 2). Ogawa serotype is more prevalent compare with Inaba with the percentage respectively 55.56% and 44.44%. While for El Tor biotype, was found more dominant compare with Classical with the percentage 88.89% for O1. All of the single isolates showed the presence of *toxR* regulator genes, but we did not find other virulence genes associated genes using primer as describe in the method.

**Table 2:** *V. cholerae* recovered from fruits and vegetables samples based on their subtypes

No	Isolate	Source	Antiserum		Polymyxin B	Multiplex PCR for virulence genes detection						
			Poly-valent	Mono-valent	Biotype	*toxR*	*ctxA*	*tcpA*	*zot*	*ace*	*ompU*	*hlyA*
	*V. cholerae* 3.21 (+ control)		Non-O1			+	+	–	–	–	–	–
1	WOR.B.2.1	West Jakarta	O1	Ogawa	El Tor	+	–	–	–	–	–	–
2	WOR.B.2.2		O1	Ogawa	Classical	+	–	–	–	–	–	–
3	AP.T.2.1	East Jakata	Non-O1			+	–	–	–	–	–	–
4	AP.T.2.2		O1	Inaba	Classical	+	–	–	–	–	–	–
5	AP.T.2.3		Non-O1			+	–	–	–	–	–	–
6	AP.T.2.4		Non-O1			+	–	–	–	–	–	–
7	AP.T.2.5		Non-O1			+	–	–	–	–	–	–
8	PI.T.3.1		O1	Inaba	El Tor	+	–	–	–	–	–	–
9	PI.T.3.2		O1	Inaba	El Tor	+	–	–	–	–	–	–
10	PI.T.3.3		O1	Ogawa	El Tor	+	–	–	–	–	–	–
11	PI.T.3.4		O1	Inaba	El Tor	+	–	–	–	–	–	–
12	PI.T.3.5		O1	Inaba	El Tor	+	–	–	–	–	–	–
13	PI.T.3.6		O1	Inaba	El Tor	+	–	–	–	–	–	–
14	PI.T.3.7		O1	Ogawa	El Tor	+	–	–	–	–	–	–
15	BL.T.4.1		Non-O1			+	–	–	–	–	–	–
16	SEL.P.1.1	Central Jakarta	O1	Ogawa	El Tor	+	–	–	–	–	–	–
17	SEL.P.1.2		O1	Inaba	El Tor	+	–	–	–	–	–	–
18	SEL.P.1.3		O1	Ogawa	El Tor	+	–	–	–	–	–	–
19	SEL.P.1.4		O1	Ogawa	El Tor	+	–	–	–	–	–	–
20	SEL.P.1.5		O1	Ogawa	El Tor	+	–	–	–	–	–	–
21	SEL.P.1.6		O1	Ogawa	El Tor	+	–	–	–	–	–	–
22	SEL.P.1.7		O1	Ogawa	El Tor	+	–	–	–	–	–	–
23	SEL.P.1.8		O1	Inaba	El Tor	+	–	–	–	–	–	–

*AP* Apple, *BL* Star fruit, *PI* Pear, *WOR* Carrot, *SEL* Lettuce

Antibiotic resistance assay showed that 4.35% of the isolates showed resistant to gentamicin, 17.39% resistant to streptomycin, 52.17% resistant to trimethoprim, 30.43% resistant to ciprofloxacin, 13.04% resistant to ampicillin, 82.61% resistant to nalidixic acid, and 91.30% resistant to polymyxin B. None of these isolates showed resistant to kanamycin (Table 3). Resistance to polymyxin B can be used to determine as biotype serogroup O1, since biotype El Tor has resistance trait for polymyxin B and otherwise for the Classical.

**Table 3:** Antibiotic resistance assay of *V. cholerae*

No	Isolates	Source	Antibiotic Disc Assay							
			CN	S	K	W	CIP	AMP	NA	PB
1	WOR.B.2.1	West Jakarta	S	S	S	R	S	S	R	R
2	WOR.B.2.2		S	S	S	R	R	S	R	S
3	AP.T.2.1	East Jakata	S	S	S	S	S	S	R	R
4	AP.T.2.2		S	S	S	S	S	S	R	S
5	AP.T.2.3		S	S	S	S	S	S	R	R
6	AP.T.2.4		S	S	S	S	S	S	R	R
7	AP.T.2.5		S	S	S	S	S	S	R	R
8	PI.T.3.1		S	R	S	S	S	R	R	R
9	PI.T.3.2		S	S	S	S	S	S	R	R
10	PI.T.3.3		S	R	S	S	S	R	R	R
11	PI.T.3.4		S	R	S	S	S	R	R	R
12	PI.T.3.5		S	S	S	R	S	S	R	R
13	PI.T.3.6		S	S	S	R	S	S	R	R
14	PI.T.3.7		S	R	S	S	S	S	R	R
15	BL.T.4.1		S	S	S	R	S	S	R	R
16	SEL.P.1.1	Central Jakarta	S	S	S	S	R	S	R	R
17	SEL.P.1.2		R	S	S	R	S	S	S	R
18	SEL.P.1.3		S	S	S	R	R	S	R	R
19	SEL.P.1.4		S	S	S	R	R	S	R	R
20	SEL.P.1.5		S	S	S	R	R	S	R	R
21	SEL.P.1.6		S	S	S	R	R	S	S	R
22	SEL.P.1.7		S	S	S	R	R	S	S	R
23	SEL.P.1.8		S	S	S	R	S	S	S	R

*CN* Gentamicin (10 µg), *S* Streptomycin (10 µg), *K* Kanamycin (30 µg), *W* Trimethoprim (5 µg), *CIP* Ciprofloxacin (5 µg), *AMP* Ampicillin (10 µg), *NA* Nalidixic acid (30 µg), *PB* Polymyxin B (300 U), *R* Resistant, *S* Susceptible

### Discussions

In this study, we utilized the MPN method for rapid estimation of *V. cholerae* prevalence in samples. On the other hand TCBS as selective medium and oxidase test were also used to ensure the presence of *V. cholerae*. The confirmation was done through PCR to distinguish between the pathogenic and non-pathogenic *V. cholerae* through detection of virulence-associated genes. Combination of MPN and PCR assay gives us rapid and sensitive detection and quantification of the presence of *V. cholerae* in fruits and vegetables, compare with conventional MPN method only.

The presence of *toxR* gene is important for regulation of other virulence-associated genes in pathogenic *V. cholerae* [13]. Therefore, this gene is used mainly to detect presence of pathogenic *V. cholerae* from samples. Pathogenic *V. cholerae* was detected in fruits from East Jakarta (21.4%) and vegetables from West Jakarta (5.26%) and Central Jakarta (6.25%). The difference in prevalence number might happened due to the distribution conditions, storage facilities, as well as sanitation in each market. In addition, some markets are located in poor and highly populated areas which is lack of access to clean water and proper sanitation. Cross-contamination may also occur since the wet market sells different types of raw meat and seafood.

For Ogawa serotype, with the percentage (55.56%) showed higher than Inaba serotype (44.44%). This results were in line with other publication found that Ogawa serotype was more prevalent than Inaba in Jakarta environment [14]. As the result of biotyping, El Tor biotype with the percentage (88.89%) was found more dominant compare with Classical biotype (11.11%). *V. cholerae* O1 El Tor is a biotype that responsible for the seventh pandemic in Celebes once.

Most *V. cholerae* found in this study belong to serogroup O1 which is known as the most often serogroup cause cholerae disease. Nevertheless, the non-O1 strain has also been reported to be involved in the emergence of a newer variant of *V. cholerae* O139 resulting in epidemic and pandemic [15]. Although non-O1 and O1 strains that we found, only had *toxR* genes, they still can cause cholera and mild gastroenteritis with unknown mechanisms [16], and there is also possibility that they might have other virulence-associated genes that had not been detected in this study Also, there is a possibility that non-pathogenic *V. cholerae* can evolve to pathogenic type due to the high possibility of horizontal virulence genes transfer [17].

### Conclusion

Combination of MPN and Multiplex PCR methods are consider sufficient to assess the prevalence and detect the presence of virulence properties of *V. cholerae.* All of *V. cholerae* found from fruits and vegetables samples were considered harmless because of the absence of virulence-associated genes. But these isolates can not be denied as they still can evoke diarrhea with unknown mechanisms or they may have other virulence-associated genes that had not been detected in this research. On the other hand the presence of *V. cholerae* in fruits and vegetables showed improper sanitation of these products. Further research was also needed to to regularly study the prevalence and detect virulence-associated genes to reduce including prevention action to reduce the presence of non-O1 and O1 serogroup and its connection to diarrheal diseases outbreaks in Indonesia.

## Limitations

The MPN method that was used in this study to enumerate *Vibrio cholerae*, might also detect other vibrio species. There is also a chance that few non-vibrio organisms are present in the enrichment medium.

## Data Availability

The datasets used and/or analysed during the current study are available from the corresponding author on reasonable request.

## Abbreviations

MPN: Most probable number
CT: Cholera toxin
Omp: Outer membrane protein
Zot: Zonula occludens toxin
